# Cerebellar hypomyelination, white matter vacuolization, and prolonged presence of atypical porcine pestivirus in pigs with congenital tremor type A-II

**DOI:** 10.1177/03009858251372559

**Published:** 2025-09-13

**Authors:** Anna Bergfeldt, Mette Myrmel, Birgit Ranheim, Frida Aae, Randi Sørby

**Affiliations:** 1 Norwegian University of Life Sciences, Ås, Norway; 2 Animalia AS, Oslo, Norway

**Keywords:** atypical porcine pestivirus, CNS, fetal infection, hypomyelination, pestivirus, pigs, transmission electron microscopy

## Abstract

Atypical porcine pestivirus (APPV) is responsible for congenital tremor (CT) type A-II in pigs, a globally distributed neurological disease, with many unresolved questions regarding its pathogenesis and pathology. This descriptive case-control study assessed the viral load of APPV and its association with lesions in the central nervous system (CNS), as piglets born with severe clinical signs of CT recovered from clinical disease. The virus was found in all pigs with CT across 3 age groups (newborn, 3-week-old, 4- to 5-month-old CT pigs) using reverse transcription-quantitative polymerase chain reaction (RT-qPCR). The highest viral load was observed in the spinal cord of newborns and in the cerebellum of older groups. No APPV was detected in control pigs. Histologic evaluation revealed variable vacuolization in the CNS white matter of CT-affected pigs, which was most prominent in the spinal cord, cerebellum, and cerebrum of newborns, and in the cerebellum and cerebrum of 3-week-old pigs. Transmission electron microscopy demonstrated hypomyelination in newborn and 3-week-old CT pigs, but myelin levels comparable to those of control pigs in 4- to 5-month-old CT pigs. This research demonstrates the prolonged presence of APPV in the CNS of pigs born with severe signs of CT. Variable white matter vacuolization and hypomyelination can be found up to 3 weeks of age, but myelin levels normalize in older pigs, suggesting a delayed myelination process. Further research is needed to confirm the primary cellular target for APPV in the CNS and to understand how the virus affects the myelination process.

Congenital tremor (CT) is a neurological disorder in newborn piglets that has been known for over a century.^23^ The syndrome is characterized by generalized tremor and is classified into 6 forms (types A-I to V, and type B) depending on etiological factors and pathology.^18^ Type B is considered idiopathic, without lesions, whereas type A is associated with hypomyelination in the central nervous system (CNS), cerebellar hypoplasia, or a combination of both. Type A-I is caused by intrauterine infection with classical swine fever virus, whereas type A-III and A-IV are hereditary in British landrace and saddleback breeds, respectively.^8,10,33^ Type A-V results from intoxication, due to metrifonate treatment in pregnant sows.^9^ These CT types were previously more common, but improved disease control, breeding systems, and drug legislation have led to their reduced occurrence. CT type A-II is likely the most frequent form of the disease today, as it is often endemic and appears as sporadic outbreaks globally. The causative agent, atypical porcine pestivirus (APPV), was discovered in 2015 using high-throughput sequencing of porcine sera and was linked to CT A-II the following year through experimental inoculation studies on pregnant sows.^3,14,20^

Tremors in CT A-II–affected piglets are considered intentional, ranging from mild to severe.^34,40^ These tremors may impair the piglet’s ability to feed and move, increasing the risk of pre-weaning morbidity and mortality due to secondary infections, starvation, or physical traumas.^3,38,40^ Over time, the clinical signs of tremor diminish, and most pigs become clinically normal by 3 to 4 months of age.^1,3,14,40^

In CT A-II, hypomyelination in the CNS is believed to be the cause of the tremors.^18^ Myelin is essential for rapid nerve conduction, and hypomyelination slows the transmission speed of electrical impulses along axons. This disruption may affect coordination between the brain, spinal cord, and muscles, leading to uncoordinated muscle responses, that manifest as intentional tremors during activity.

Studies of the pathogenesis and pathology of CT A-II have mostly been conducted on newborn piglets, with inconsistent pathological findings. These findings include combinations of mild hypomyelination and/or mild to moderate vacuolization in the spinal cord or cerebellum, but an absence of lesions has also been described.^16,32,38–40,43,51^

Hypomyelination is considered a developmental disorder which can be either delayed or irreversible. Piglets affected by CT A-II eventually recover from the clinical signs of disease, suggesting a transient nature of hypomyelination, although this has yet to be confirmed.

Following infection, APPV spreads to all organ systems.^3,38,40^ Within the CNS, the highest virus levels have been detected in the cerebellum in CT-affected newborns.^12,25,38,39^ Although APPV crosses the blood-brain barrier, signs of inflammation are rarely described in CT A-II cases.

Many aspects of the pathogenesis and pathology of APPV-induced CT remain unknown. Gaining a deeper understanding of APPV’s tissue tropism and CNS pathology could provide valuable insights that support the development of new approaches to improve pig welfare and health, as well as future studies in the field.

The objectives of this study were (1) to describe and quantify lesions in pigs born with severe CT, (2) to map the concurrent presence of APPV in the CNS, and (3) to assess the variations in APPV load and CNS lesions as piglets with CT recover from clinical signs of the disease.

## Material and Methods

### Ethical Statement

The study was conducted in accordance with Norwegian legislation for animal welfare and approved by the Norwegian Food Safety Authority (FOTS ID: 25780).

### Sampling

A field study was performed in 2021–2022, involving 5 pig herds in eastern central Norway. Four herds experienced natural outbreaks of CT, whereas one herd, with no history of CT, served as a negative control. In a parallel study on clinical signs, all pigs were regularly examined from birth to slaughter age.^1^ This article is part of a larger investigation into CTs in pigs, and detailed information about the production form and breeds used across all herds is published by Aae et al.^1^

For the present case-control study, 3 age groups were included: newborns (2- to 4-days-old), 3-week-old pigs, and pigs aged 4 to 5 months. Samples were collected from 5 CT pigs and 5 controls in each age group. Supplemental Table S1 provides a summary of the herd origin for pigs selected for pathology, and Supplemental Table S2 includes details on herd type and breeds. The included CT-affected piglets were born with severe tremors, defined as intense muscle contractions that disturbed voluntary movements. Tremors increased during activity or stress, decreased during rest, and were not visible during sleep. Further details on clinical presentation during the CT outbreaks are published by Aae et al.^1^ No other signs of disease were observed in the pigs at the time of inclusion. One CT herd was excluded from this study due to concurrent issues with arthritis and dermatitis.

### Necropsy

Euthanasia and necropsies were performed on-site at the respective farms for newborn and 3-week-old pigs, and within the premises of the Faculty of Veterinary Medicine, at the Norwegian University of Life Sciences (NMBU), for the 4- to 5-month-old pigs. The oldest pigs were transported to NMBU a minimum of 1 day ahead of the necropsy. The newborns and 3-week-old pigs were anesthetized with an intramuscular injection of zolazepam, tiletamine, and xylazine (Zoletil Vet/ Zoletil forte Vet; Virbac, Denmark; Rompun, Elanco, Denmark), and the 4- to 5-month-old pigs with a combination of ketamine, xylazine, and midazolam (Ketador, Richter Pharma, Austria; Rompun, Elanco, Denmark; Midazolam Accord, Accord Healthcare, Spain). All pigs were euthanized by an intracardiac injection of pentobarbital (Euthasol vet., Le Vet, The Netherlands). Following euthanasia, all pigs were immediately subjected to necropsy and tissue sampling. Body weights were recorded before the necropsy, and the weight of the brains were recorded directly after their removal.

During necropsy, tissue specimens from the CNS and nerves (N. ischiadicus and N. ulnaris) were obtained as promptly as possible, followed by samples from internal organs (myocardium, lung, liver, kidney, ileum, jejunum, and colon), lymphatic tissue (tonsils, thymus, ln. submandibularis, and ln. inguinalis superficialis), skeletal muscle (M. rectus femoris), and reproductive organs (testicles or ovaries). In the newborns and 3-week-old pigs, a pair of curved scissors was used to remove the cranium, while a combination of a hand-held saw and a small mallet and chisel were used in the oldest pigs. Six coronal sections were sampled from the brain (at the level of the motor cortex, thalamus, midbrain, cerebellum, pons, and medulla oblongata), and 3-to-4 sections were sampled from random areas of the cervical, thoracic, and lumbar parts of the spinal cord. A description of stereotaxic locations and areas involved in motor functions in all sampled sections from the CNS is available in Supplemental Table S3. Samples for histology were fixated in 10% neutral-buffered formalin and care was taken to fixate all brain tissues for similar lengths of time (10–14 days). Samples for virus detection were preserved in RNA*later* (Thermo Fisher, Massachusetts, USA) for 24 hours at room temperature, before storage at −80°C until further processing. For ultrastructural studies, samples were fixed in 2.5% glutaraldehyde in 0.1 M Sorensen phosphate buffer pH 7.4, cut in 1 to 2 mm^3^ cubes, and stored at room temperature for 24 hours. The samples were then placed into 0.1 M Sorensen phosphate buffer pH 7.4 and stored at 4°C until processing.

### RNA Isolation

The CNS areas selected for RNA isolation were those with the most lesions observed by light microscopy. To evaluate the APPV load in CNS, 19 mg of thawed tissue from the cerebrum, cerebellum, and thoracic spinal cord were homogenized according to the RNeasy protocol for lipid rich tissue from Qiagen. In short, Qiazol Lysis reagent (500 μl) and a 5-mm steal bead was added to the sample in a 2-ml tube, followed by homogenization at 20 Hz until the sample was completely homogenized (FastPrep-24). Next, 100 μl of chloroform was added, and the samples were centrifuged at 4°C for 3 minutes at 14,000 rpm (Thermo Scientific Fresco 21 microcentrifuge). RNA was isolated from 200 μl of the upper aqueous phase using the QIAsymphony RNA kit (Qiagen, Hilden, Germany) and the Qiagen QIAsymphony SP, with an elution volume of 50 μl. The RNA quality was evaluated using an Epoch microplate spectrophotometer (BioTek, Winooski, VT, USA), and total RNA was quantified using the Qubit RNA High Sensitivity assay kit (Invitrogen, Massachusetts, United States) on an Invitrogen Qubit 4 Fluorometer.

### Reverse Transcription-Quantitative Polymerase Chain Reaction (RT-qPCR)

An evaluation of APPV load in CNS was performed using the RNA UltraSense One-Step Quantitative RT-PCR System kit (Thermo Fisher). The analysis targeted the coding sequence of the non-structural protein NS5B using the forward primer APPV-NS5B-303F, reverse primer APPV-NS5B-385R, and probe APPV-NS5B-336-FAM, as previously described.^6^ The RT-qPCR was run on an AriaMx Realtime PCR System (Agilent Technologies) with 2 μl of RNA, 16 μl of Master Mix, 400 nM of each primer, and 200 nM of the probe, using the following cycling conditions: 50°C for 30 minutes, 95°C for 2 minutes, [95°C for 15 seconds, 56°C for 40 seconds] × 45. The results from the RT-qPCR were visualized in Aria MX software.

### Histology and Immunohistochemistry

For morphological evaluation of tissue samples, standardized histologic processing techniques were employed, including paraffin wax embedding, sectioning to a 4-μm thickness, and staining with hematoxylin and eosin. Histologic assessments were carried out on a brightfield microscope (Nikon Eclipse Ci), or via digitally scanned slides (Philips, IntelliSite Ultra-fast Scanner). A stereotaxic atlas of the porcine brain was referenced to identify regions associated with motor functions.^19^

Cerebellar sections from 3 CT pigs and 3 controls from the newborn and 3-week-old groups were stained with luxol fast blue and cresyl violet to detect myelin.

The cellular composition of gliosis in the oldest CT pigs was evaluated using immunohistochemistry. Slides from four out of five 4- to 5-month-old CT-affected pigs, with representative changes in cerebrum and spinal cord, were assessed. Anti-GFAP antibodies (Prod. nr: Z033429-2, 1:500 dilution; Agilent Dako, Glostrup, Denmark) were used as marker for astrocytes, whereas anti-IBA1 antibodies (Prod. nr: 019-19741, 1:1000 dilution; FUJIFILM Wako Pure Chemical Corporation, Osaka, Japan) were used as marker for microglia and macrophages. Cerebellar slides served as positive controls for GFAP and IBA1, whereas negative controls were obtained by incubating cerebellar slides without the primary antibody.

The formalin-fixed paraffin-embedded slides were deparaffinized in xylene, rehydrated in ethanol, and rinsed in distilled water. For IBA1 slides, proteolytic antigen retrieval was performed in trypsin buffer (pH 8.0) at 37°C for 40 minutes. Endogenous peroxidase activity was inhibited by incubation with hydrogen peroxide for 10 minutes (Peroxidazed 1, Biocare Medical, Pacheco, CA, USA), and non-specific binding was blocked with a 10-minute incubation using Background Sniper (MACH 1 Universal Polymer Detection; Biocare Medical). The primary antibodies were diluted in Da Vinci Green Diluent (BioCare Medical) and incubated for 30 minutes. A polymer-based detection system, incorporating secondary antibodies and HRP attached to a polymer backbone, was used for 30 minutes (Universal HRP, MACH1; Biocare Medical). The AEC Romulin Chromogen Kit (BioCare Medical) was applied to visualize the polymer-bound antigens. All incubations were conducted at room temperature and washing with phosphate-buffered saline was performed between steps, unless otherwise specified. Finally, the slides were counterstained with Mayer’s hematoxylin, dehydrated, cleared in xylene, and coverslipped.

### Vacuole Quantification

Quantification of vacuole density (*n*/mm^2^) was performed using the bioimage analysis software QuPath (Supplemental Table S4).^5^ Regions of interest (ROIs) were evaluated in sections from the cerebrum (large white matter tracts, at the level of the motor cortex), cerebellum (the unbranched body of the white matter in the lateral hemisphere of the cerebellum), and spinal cord (lateral and ventral funiculi, bilateral). ROIs were selected based on a high degree of vacuolization observed under brightfield microscopy. To enhance comparability and reduce variations in vacuole distribution patterns within and between ROIs and individuals, a large examination area was chosen. For each pig, one section per ROI was examined, analyzing the complete area of the ROI within that section. An increase in vacuole density was defined as values > mean vacuole density + 3 standard deviations in the age matched control group, and the same value was used to describe the overall increase in vacuolization.

### Transmission Electron Microscopy

Ultrastructural evaluation of the unbranched body of the white matter in the lateral hemisphere of the cerebellum was based upon high APPV load and white matter vacuolization. Sections within the subfoliar white matter at the peripheral border of the unbranched body of white matter were selected by convenience for further processing. The samples were washed with 0.1 M cacodylate buffer (Na (CH3)2AsO2) for 3 times for 10 minutes before further fixation and staining with 1% osmium tetroxide in 0.1 M cacodylate buffer for 1 hour at 4°C. Dehydration was performed with a series of increasing concentration of acetone (50% for 3 × 10 minutes, 70% for 2 × 10 minutes, 90% for 2 × 10 minutes and 100% for 3 × 20 minutes), before infiltration with a 1:1 mixture of acetone and Epon resin for 1 hour followed by 2 × 30 minutes of incubation in pure Epon resin. Finally, the samples were embedded with Epon resin in capsules and polymerized at 60°C for 48 hours.

Semi-thin sections (500 nm) were cut and stained with toluidine blue for brightfield microscopy to select suitable areas for examination using Transmission electron microscopy (TEM), before trimming and ultra-thin sectioning (70 nm). The sections were placed on a grid and contrasted with 4% uranyl acetate and Reynolds lead citrate before the TEM study, which was performed on a JEOL, JEM 2100-Plus electron microscope operated at 80 kV and connected to a TVIPS XF416 Camera and EM Menu 5 software.

### Ultrastructural Evaluation

**Figure 1.**
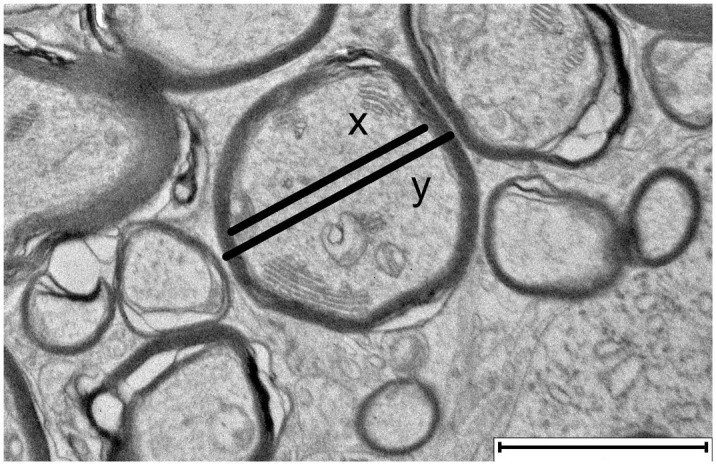
Transmission electron microscopy image of cerebellar white matter, showing transverse cross-sections of myelinated nerve fibers. The g-ratio describes the thickness of the myelin sheath in relation to the axon diameter and is calculated as the ratio of the axon diameter (x) to the diameter of the entire myelinated nerve fiber (y). Scale bar = 2 μm.

Areas with a high density of transversely cut nerve fibers, free of interfering structures like capillaries or glia cells, were selected for imaging. For each individual, 10 to 20 images were captured at 4000× magnification. To calculate the g-ratio, which represents the relationship between axon diameter and the total diameter of the myelinated nerve fiber, measurements of axon and nerve fiber thickness were conducted on 100 nerve fibers per individual, as previously described, using QuPath software (Fig. 1).^4,49^ Degenerative changes in white matter were analyzed in ten images from each pig. The analysis included a qualitative evaluation of myelin disruption in individual myelinated axons, with lamellar unraveling categorized as mild (<25%), moderate (25%–75%), or severe (>75%). In addition, the presence of intra- and extra-axonal myelin debris, intra-axonal vacuoles, and myelin ballooning was assessed.

### Statistical Analysis

All analyses were performed in Jamovi 3.2.18. Descriptive statistics were presented as mean values at group level, with 95% confidence intervals or minimum-maximum values, as well as standard deviations. Pearson’s *r*-coefficient was used for correlation testing. Graphical presentation of RT-qPCR results and g-ratios were created in BioRender.com.

### Data Availability

The analyzed data are either available in the Supplemental Materials or can be made available on request.

## Results

### Virus Load and Distribution in the CNS

**Figure 2.**
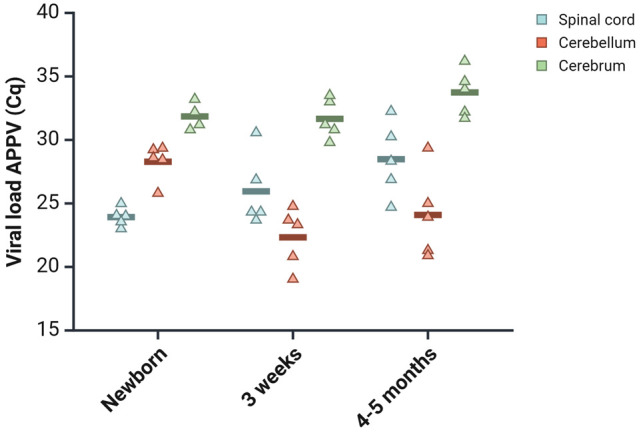
Evaluation of atypical porcine pestivirus (APPV) levels in the central nervous system (CNS), ranging from newborn to those aged 4 to 5 months, as determined by reverse transcription-quantitative PCR. The jitter plot displays Cq values for APPV across various CNS regions in pigs affected by CT across different age groups. Each point represents the Cq value of an individual pig, with the horizontal line indicating the mean value for each group.

The load of APPV in cerebrum, cerebellum, and thoracic spinal cord was examined using RT-qPCR. All CT-affected pigs were APPV-positive in all 3 CNS areas, but APPV was not found in any of the control pigs. The mean viral load of the spinal cord was highest in the newborns (lowest Cq value), while cerebellum held the highest mean virus levels in 3 weeks and 4 to 5 months. The virus loads in cerebrum remained relatively constant from birth to 4 to 5 months of age, and this region had the lowest viral loads across all 3 age groups (highest Cq values) (Fig. 2 and Supplemental Table S5).

### Gross and Histopathologic Findings

None of the pigs had gross pathologic changes of significance; however, incidental findings like skin lesions and kidney cysts were observed in isolated cases. The newborns had varying amounts of milk in their stomach. Neither the brains, nor the spinal cords had macroscopic lesions, and the relative brain-to-body weight ratios did not differ between control- and CT-affected pigs.

Histologically, all CT-affected newborns (5/5) had increased vacuolization within 2 to 3 segments of the spinal cord (cervical, thoracic, lumbar). Most newborn and 3-week-old CT-affected pigs also showed a variable increase of white matter vacuolization in cerebrum and cerebellum compared with controls. All but one (9/10), showed increased vacuolization in the inferior cerebellar peduncle. The vacuolization observed in the 4- to 5-month-old CT pigs was similar to controls. A summary of the stereotaxic location of hematoxylin and eosin-stained sections with white matter vacuolization is provided in Supplemental Table S3.

The vacuoles were oval or round, mostly empty, but were occasionally filled with eosinophilic, amorphous material, or cellular components. They were distributed multifocally within the white matter, typically appearing as isolated structures. However, in pigs with high degrees of vacuolization, 2 or more vacuoles could be seen seemingly fused together, or separated by a thin septum.

**Figure 3.**
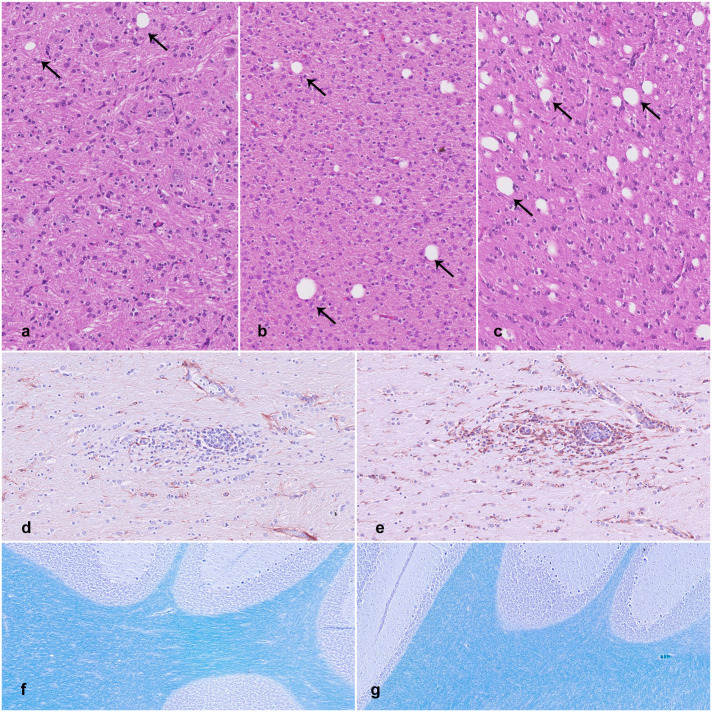
Central nervous system lesions associated with congenital tremor type A-II. (a–c) Variation in cerebellar white matter vacuole density across newborn pigs affected by congenital tremor (CT). Hematoxylin and eosin (HE). (a) Few scattered vacuoles, comparable to control group density. (b) Mild to moderate increase in vacuolization, with a density that is 2 to 8 times higher than controls. (c) Severe vacuolization, with a density that is 18 times higher than controls. (vacuoles, arrows). (d, e) Large white matter tracts, cerebrum. Mild, focal, perivascular gliosis in a 4- to 5-month-old CT-affected pig. (d) GFAP immunolabeling for astrocytes. (e) IBA1 immunolabeling for macrophages and microglia. (f, g) Luxol fast blue stain for myelin in cerebellar sections. Luxol fast blue-cresyl violet stain. (f) Three-week-old control pig. (g) Three-week-old CT pig. No differences in stain intensity for myelin are observed.

Mild focal to multifocal gliosis could be found in the oldest CT-affected pigs, mostly in the white matter (5/5) or perivascular regions (3/5), in different parts of CNS. The gliosis mainly consisted of cells from the monocytic lineage (IBA1-positive) and was interpreted as microgliosis (Fig. 3d, e). Positive and negative controls immunolabeled as expected.

Differences in myelin levels between CT-affected pigs and controls were not detected using luxol fast blue-cresyl violet staining (Fig. 3f, g). However, the myelin staining in newborns appeared slightly lighter compared with the 3-week-old pigs.

No lesions could be found in gray matter in any of the pigs.

Mild to moderate, interstitial, histiocytic pneumonia was a common incidental finding in the control pigs (14/15) and was also seen in the 3-week-old CT pigs (4/5). Poor air quality, due to high ammonia concentrations and dust, was considered a likely cause of the histiocytic pneumonia, as the lung lesions had a uniform, diffuse distribution and was seen in several age groups.

### Vacuole Density

The increase in vacuole density (n/mm^2^) in the lateral and ventral funiculi of the spinal cord was highest in the thoracic and lumbar regions in all newborn CT pigs, with a 2- to 15-fold increase compared with the control group.

In the cerebellum, one newborn and two 3-week-old pigs (3/10) showed no increase in vacuole density compared with controls (Fig. 3a). Many pigs (6/10) had a mild-to-moderate increase, with a 2- to 8-fold rise in vacuoles (Fig. 3b). One newborn (1/10) demonstrated a severe increase, with vacuole density 18 times higher compared with controls (Fig. 3c, Table 1). In the cerebrum, two newborns and three 3-week-old CT pigs showed a 2- to 6-fold increase in vacuole density. All piglets with the highest degree of vacuolization originated from the same herd. There was no correlation between viral load and vacuole density.

**Table 1.** Summary of white matter vacuole density in hematoxylin and eosin-stained CNS sections from pigs affected by congenital tremors (CT) and controls.

		Vacuole Density (n/mm^2^)					
		CT-Affected Pigs			Controls		
Age	Area	Mean	Min-Max	SD	Mean	Min-Max	SD
Newborn	Cb	1.96	0.00–6.71	2.85	0.45	0.07–0.80	0.32
	Cbm	6.95	0.43–25.5	10.5	0.53	0.25–0.93	0.30
	C.sc	1.96	0.31–5.50	2.11	0.16	0.10–0.24	0.10
	T.sc	8.49	3.43–17.32	5.66	0.45	0.00–1.30	0.50
	L.sc	2.46	0.99–6.44	2.27	0.14	0.00–0.22	0.08
3 wks.	Cb	8.31	0.33–24.2	9.74	1.24	0.52–3.03	1.02
	Cbm	4.74	0.34–10.00	4.42	0.33	0.09–0.88	0.32
	T.sc	0.77	0.24–1.49	0.47	0.10	0.00–0.39	0.17
4-5 mos.	Cb	0.72	0.46–1.36	0.37	0.64	0.15–1.36	0.47
	Cbm	0.38	0.12–0.74	0.26	0.29	0.27–0.35	0.04
	T.sc	0.25	0.11–0.43	0.19	0.10	0.00–0.27	0.12

Vacuole density was quantified using QuPath software.

Abbreviations: CNS, central nervous system; wks, weeks; mos, months; Cb, cerebrum; Cbm, cerebellum; C.sc, cervical spinal cord; T.sc, thoracic spinal cord; L.sc, lumbar spinal cord; SD, standard deviation; n, number of vacuoles.

### Transmission Electron Microscopy

**Figure 4.**
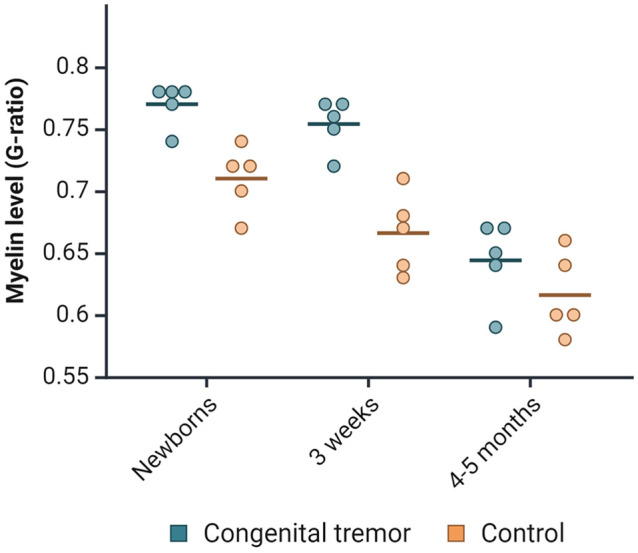
Distribution of g-ratios in newborns, 3-week-old, and 4- to 5-month-old pigs affected by congenital tremors compared with controls. Each point in the jitter plot represents an individual pig, with the horizontal lines indicating the mean value for each group.

The ultrastructure of cerebellar white matter from the lateral hemisphere was examined with TEM. The CT-affected newborns and 3-week-olds had increased mean g-ratios, indicating hypomyelination. However, the thickness of myelin sheaths in the 4- to 5-month-old CT pigs was comparable to that of the controls (Fig. 4). The newborn CT-affected piglets had a mean g-ratio of 0.77, compared with 0.71 in the controls. In the 3-week-old CT pigs, the difference in mean g-ratio between the groups was higher, with 0.75 in CT pigs compared with 0.67 in the control group. The differences in mean g-ratios equaled an average of 21% less myelin in CT newborns and 26% less in the 3-week-old CT pigs compared with healthy controls. However, one newborn and one 3-week-old CT pig (2/10) showed g-ratios comparable to the control pigs. These CT pigs also had a low density of white matter vacuolization in cerebrum and cerebellum. Still, the newborn had increased vacuolization in thoracic and lumbar spinal cord, as well as in the inferior cerebellar peduncle.

The reduced thickness in myelin sheaths was consistent across all sizes of myelinated nerve fibers. The diameter of myelinated nerve fibers in CT pigs was skewed to the right compared to controls, with a longer right-side tail.

**Figure 5.**
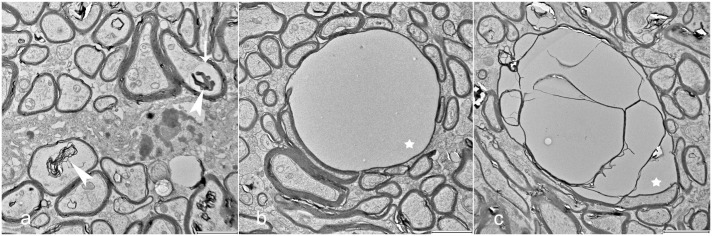
Degenerative changes in myelinated nerve fibers within cerebellar white matter. Transmission electron microscopy. (a) Myelin debris (arrowheads) and an intra-axonal vacuole (arrow). (b, c) Myelin vacuoles (stars), including simple vacuoles with the axon positioned as a thin crescent at the side, and large vacuoles containing multiple internal units. Scale bars = 2 μm in (a, b), and 3 μm in (c).

Indicators of myelin degeneration in individual axons, such as severe myelin disruption, myelin ballooning, intra- and extra-axonal myelin debris, and intra-axonal vacuoles, were more prevalent in the CT-affected pigs across all age groups compared with healthy controls (Fig. 5a). However, the overall presence of these changes was assessed as mild. Mild to moderate lamellar disruption within the myelin sheaths was found at similar levels in both control and CT-affected pigs, likely due to fixation artifacts, which is a common phenomenon when working with nervous tissue (Supplemental Figure S1).^49^ The vacuoles observed using light microscopy were located within myelin sheaths, but it was not possible to determine whether they were intra- or extracellular (Fig. 5b). In the CT pigs, some vacuoles were separated into multiple compartments of various sizes, without an obvious center (Fig. 5c). A positive correlation was found between the g-ratio and the degree of myelin vacuolization (*r* = .73, *P* = <.001).

## Discussion

This study aimed to examine the variations in APPV load and CNS pathology in pigs born with severe CT type A-II, throughout their recovery from clinical disease. We found that APPV can persist in CNS for 4 to 5 months, while hypomyelination and variable myelin degeneration can be detected until 3 weeks of age.

A case-control study was designed to allow for comparisons of the neonatal myelinization process in CT pigs with healthy control pigs of the same age. All CT-affected pigs were born with an equal degree of severe and generalized, intentional tremors. Neither clinical examination, nor histological evaluation indicated any co-existing health problems in the pigs that could potentially bias the evaluation of CT.

Our results suggest that CNS lesions evolve during recovery from severe CT A-II. White matter vacuolization in the spinal cord was present only in newborns. In contrast, both cerebellar hypomyelination and white matter vacuolization in cerebrum and cerebellum were observed in both newborns and 3-week-olds, but these lesions were absent in pigs aged 4 to 5 months. The highest APPV load was detected in the cerebellum of 3-week-old and 4- to 5-month-old CT pigs; however, myelin levels and organization had normalized in the older age group. The lack of correlation between APPV load and lesion severity indicates that postnatal CNS lesions result from interference with the myelination process earlier in gestation, rather than an ongoing response to the virus.

Compared with earlier reports, our study generally observed a higher degree and wider distribution of vacuolized white matter in CT newborns.^16,39,40,45^ Previous studies have described white matter vacuolization as absent or mild, with only a small number of vacuoles.^16,38–40,43,45,51^ The more pronounced vacuolization observed in our study might be explained by the exclusive inclusion of pigs with severe generalized tremors, whereas the severity of tremor in pigs in referenced studies was not specified.

The tremors in CT A-II vary in severity, and our study demonstrated a correlation between white matter vacuolization and cerebellar hypomyelination, which may indicate that severe clinical signs could be associated with increased hypomyelination as well as myelin degeneration.^1,3,14^ This theory is supported by a 1976 study of experimentally induced CT A-II, conducted before the discovery of APPV, which found a correlation between spinal cord myelin deficiency and tremor severity in pigs through histologic analysis.^32^ In addition, the same study noted that hypomyelination of the spinal cord could persist up to four weeks in some CT pigs, a finding consistent with our results.

In the present study, spinal cord vacuolization was only found in newborn pigs and not in the 3-week-old group. It would have been interesting to quantify myelin levels in the spinal cord in addition to cerebellum to determine any correlation between them. We also detected increased vacuolization in the inferior cerebellar peduncle in nearly all CT newborns and 3-week-olds. This structure connects the cerebellum to the medulla oblongata and facilitates both efferent and afferent nerve signals involved in balance, coordination, and correction of voluntary motor activities.^11,22^ These findings might shed light on the correlation between the pathology and clinical signs in CT A-II, as well as be used for diagnostic purposes.

Earlier reports of hypomyelination in CT A-II have been qualitative, and luxol fast blue stain for myelin has been used to visualize this condition in the spinal cord.^2,3,16,38–40^ Previous studies employing this staining method on cerebellar white matter have not detected hypomyelination, which is in line with our results.^40,43^ Two studies have used TEM to evaluate samples from a newborn CT piglet and a control, both of which detected mild myelin disruption in the CT pigs.^40,43^ The first study also found swollen mitochondria in cerebellar oligodendrocytes without hypomyelination, whereas the second reported mild hypomyelination, along with myelin disruption and breakdown in cerebellar white matter and the brain stem.^40,43^ Neither study calculated g-ratios to estimate myelin thickness.

To our knowledge, the present study is the first larger quantitative TEM analysis of CT A-II. Our observation of a mild increase in myelin disruption across all CT age groups aligns with previous studies. However, the 2-dimensional nature of TEM images might complicate the determination of whether intra- and extra-axonal myelin fragments represent debris or artifacts resulting from localized loosening of myelin lamellae in adjacent regions. Regardless of the underlying cause, the higher incidence observed in CT-affected pigs may indicate more pronounced interlamellar instability compared to controls.

In addition, we identified thinner myelin sheaths and quantified higher g-ratios in 8 out of 10 newborns and 3-week-old CT piglets. Our evaluation of cerebellar white matter using TEM was limited to small areas from each pig. Expanding the sampling to include more regions of CNS white matter might have revealed hypomyelination in the remaining 2 CT pigs, given that the distribution pattern of cerebellar hypomyelination in CT A-II remains uncertain. Interestingly, Buckley and colleagues identified a multifocal distribution pattern of APPV in newborn CT pigs in the granular and molecular layers of cerebellum, whereas a more diffuse distribution was seen in 11-month-old CT boars.^12^ These findings suggest that variations in myelin thickness may be influenced by the distributional differences of APPV within the tissue. While in situ detection of APPV could have provided further insights into the virus distribution and its association with hypomyelination, this method was not prioritized in the current study.

Hypomyelination and white matter vacuolization have been reported to occur at the same time in CT A-II.^16,39,40^ However, there are also cases where white matter vacuolization was the only pathological change, or no lesions were observed, as seen with bright field microscopy.^28,45,51^ The previously mentioned TEM study failed to detect hypomyelination using luxol fast blue stain for myelin, as well as electron microscopy on a CT newborn.^43^ It is difficult to determine whether these cases truly lacked concurrent hypomyelination in the CNS or if it could have been detected in a more extended diagnostic investigation.

The normalized thickness of myelin sheaths observed in pigs aged 4 to 5 months in the present study indicates a delayed myelination process. Such a delay has been proposed as a possible explanation for the recovery from CTs associated with other pestiviruses, such as border disease virus in sheep, and classical swine fever virus infection in CT A-I.^10,27,46^ However, in these conditions, the impact on myelination appears to be more severe than for CT A-II, with signs of demyelination and remyelination, in addition to hypomyelination. CT resulting from in utero infection with bovine viral diarrhea virus is considered less common than other outcomes, with diffuse hypomyelination as the principal histopathological findings, sometimes accompanied by gliosis.^7,29,31,37^ Further investigation into oligodendrocyte activity and gene expression using immunohistochemistry or transcriptomics, respectively, could provide a deeper understanding of the postnatal effects of APPV on the myelination process.

Our finding of prolonged high viral loads in the cerebellum is in line with previous reports.^12,25^ Although histopathologic lesions have not been identified in cerebellar gray matter, APPV has been localized to the internal granular cell and molecular layers using immunohistochemistry and RNA in situ hybridization, in both newborns and 11-month-old boars with CT.^12,25,38^ So far, APPV has not been found in any supportive cells within the white matter or in Purkinje cells. However, it has been detected along the axons of granular cells and in nerve fibers in the cerebellar medulla.^25^ A potential decrease in granular cell activity could impact the myelination process, as nerve fiber signaling has been shown to positively affect myelin production.^21,44^

The absence of observable leukocyte infiltration and perivascular cuffs, despite the presence of APPV in the CNS, is consistent with earlier histologic descriptions of newborn CT A-II pigs.^16,28,38,43^ To evade the adaptive immune response, viruses must infect the porcine fetuses before they become immunocompetent, which is by day 70 to 80 of gestation.^35,42^ Pestiviruses are also known to evade the innate immune system, by producing the protein E^rns^, which blocks the expression of key cytokines and interferons.^15,26^ The mild, multifocal microgliosis observed in the oldest CT pigs may represent a clean-up process secondary to myelin disruption or a mild chronic reaction to the virus. Although mild ultrastructural myelin disruption was found in all 3 age groups of CT pigs via TEM, gliosis was present only in the oldest, suggesting that clearance of myelin debris might not be the sole reason for the microgliosis. Whether these lesions are related to myelin disruption or to other unrelated factors needs to be further investigated.

APPV was identified as the likely cause of the tremors, as all CT pigs tested positive for the virus and other potential causes for CT are considered unlikely under Norwegian conditions. Norway is officially free from classical swine fever, the use of metrifonate in food producing animals is banned, and the breeds genetically predisposed for CT types A-III and A-IV are not in use.

Regarding other viruses associated with CT A-II, the porcine circovirus-like virus P1 and lindavirus have only been found in localized outbreaks in China and Austria, respectively.^24,50^ In addition, the previously proposed association between CT A-II and porcine circovirus type 2 or 3 is now considered controversial due to inconsistent results.^13,41^ Moreover, no lesions consistent with systemic porcine circovirus infection were observed in lymph nodes or other tissues at macro- or microscopic levels.

Among other potential causes for tremors in young piglets, hypoglycemia and hypothermia were ruled out in the newborns, as they all showed signs of feeding and maintained a body temperature within the normal range.^1^ In addition, tremor associated with such conditions typically persist during rest, while in the present study, tremors were only observed during activity.^1,48^ Infectious agents like African swine fever virus, pseudorabies virus, and highly pathogenic strains of porcine reproductive and respiratory syndrome virus have also been associated with tremor conditions in young pigs. However, Norway is declared free from these pathogens, and they are typically linked to more severe, systemic disease presentations with higher mortality rates compared with those observed in outbreaks of APPV-induced CT.^30,36,47^ Finally, various intoxications and nutritional deficiencies can also cause neurological signs, though generally not congenital and usually occurring as part of a broader range of clinical and histopathological findings.

Although all herds in the present study followed standard Norwegian production practices, field data generally show more variation than experimental studies, reflecting differences in herd management, genetics, and health status. While such factors may have influenced the results, a high degree of similarities between herds made it difficult to identify any possible influencing factors. A larger sample size and more herds could have provided more robust results. Also, as infection earlier during gestation has been related to increased spinal cord hypomyelination in CT A-I, a controlled timing of maternal infection in an experimental setting might reduce litter-to-litter variation in myelin changes.^17^

This study describes and quantifies the effects of APPV on the CNS in pigs born with CT A-II as they recover from clinical disease. The findings of APPV persistence in the cerebrum, cerebellum, and spinal cord, along with a temporary cerebellar hypomyelination and variable myelin vacuolization up to 3 weeks of age, contribute to our understanding of the pathogenesis of CT A-II. Future research will hopefully elucidate how APPV affects the CNS in utero at the time of infection and how those events disrupt the myelination process.

## Supplemental Material

sj-pdf-1-vet-10.1177_03009858251372559 – Supplemental material for Cerebellar hypomyelination, white matter vacuolization, and prolonged presence of atypical porcine pestivirus in pigs with congenital tremor type A-II Supplemental material, sj-pdf-1-vet-10.1177_03009858251372559 for Cerebellar hypomyelination, white matter vacuolization, and prolonged presence of atypical porcine pestivirus in pigs with congenital tremor type A-II by Anna Bergfeldt, Mette Myrmel, Birgit Ranheim, Frida Aae and Randi Sørby in Veterinary Pathology
