# Supplementary material for: Cerebellar hypomyelination, white matter vacuolization, and prolonged presence of atypical porcine pestivirus in pigs with congenital tremor type A-II
Source: Vet Pathol. 2025 Sep 13;63(2):244–54. doi: 10.1177/03009858251372559 (PMC12882986; doi:10.1177/03009858251372559)
Supplement: sj-pdf-1-vet-10.1177_03009858251372559 – Supplemental material for Cerebellar hypomyelination, white matter vacuolization, and prolonged presence of atypical porcine pestivirus in pigs with congenital tremor type A-II [file sj-pdf-1-vet-10.1177_03009858251372559.pdf]

Supplemental Materials

Cerebellar hypomyelination, white matter vacuolization, and prolonged presence of atypical porcine pestivirus in pigs with congenital tremor type A-II

Anna Bergfeldt, Mette Myrmel, Birgit Ranheim, Frida Aae, Randi Sørby

Supplemental Table S1. Summary of herd origin and number of pigs selected for pathology and atypical porcine pestivirus detection, categorized by age group.

Herd	Status	Newborn	3-week-old	4- to 5-month-old
A	CT outbreak	2	-	2
B	CT outbreak	2	3	2
C	CT outbreak[a]	-	-	-
D	CT outbreak	1	2	1
K	Control	5	5	5

[a]This herd was excluded from this study due to concurrent problems with arthritis and dermatitis

Abbreviation: CT, congenital tremor

Supplemental Table S2. Production form and breed information for the breeds included in the study.

Herd	Herd type	Sow breed	Insemination breed
A	Farrow-to-finish	TN70	Duroc
B	Farrow-to-finish	TN70	Duroc
		Norwegian Landrace	Norwegian Landrace
C	Farrow-to-nursery	TN70	Duroc
D	Farrow-to-finish	TN70	Duroc
		Norwegian Landrace	Norwegian Landrace
K	Farrow-to-nursery	TN70	Hampshire

Supplemental Table S3. Summary of central nervous system sections evaluated with histology, detailing stereotaxic locations and areas related to motor functions. The assessment covers whole sections and highlights regions with increased vacuolization in newborn and 3-week-old pigs affected by congenital tremor (CT).

Evaluated section	Stereotaxic location[1]	Areas related to motor function	Increased vacuolization
Section 1: Cerebrum	A 14.5 mm	Motor cortex.	-
		Large white matter tracts	+ - +++
Section 2: Thalamus	A 7.0 mm	Large white matter tracts	+ - +++
		Thalamic nuclei	-
Section 3: Thalamus	A 5.0 mm	Large white matter tracts	+ - +++
		Thalamic nuclei	-
		Substantia nigra	-
Section 4: Midbrain, pons	Diagonal cut: Dorsal P 8.00 mm Ventral A 2.00 mm	Red nucleus	-
		Substantia nigra	-
Section 5: Cerebellum	P 14.00 mm	White matter	+ - +++
		Deep cerebellar nuclei	-
		Grey matter (Purkinje cells, granular cell layers, molecular layer)	-
		Inferior cerebellar peduncle	+ - +++
		Olivary nucleus	-
Section 6: Medulla oblongata	P 17-18.50 mm	Reticular formation	-
Spinal cord	Variable[a]	White matter (dorsal, lateral and ventral funiculi)	+ - +++

[1] Felix B, Leger ME, Albe-Fessard D, Marcilloux JC, Rampin O, Laplace JP. Stereotaxic atlas of the pig brain. *Brain Res Bull.* 1999;49(1-2):1-137.

[a] Three to four sections were sampled from each of the cervical, thoracic, and lumbar segments of the spinal cord.

Supplemental Table S4. Settings used in QuPath software to quantify vacuole density in HE-stained CNS sections from pigs affected by congenital tremor (CT) and controls.

QuPath function	Aim/description
Polygonal tool	Manual selection of predefined region
Create Thresholder	Automatic selection of vacuoles:
- Resolution	Very high
- Channel	Average channels
- Prefilter	Gaussian
- Smoothing sigma	5.5
- Threshold	200-210 [a]
- Above threshold	Positive
- Below threshold	Negative
- Region	Any annotation
- Minimum object size	100 μm^2 [b]
- Split objects	Yes [c]
Set class	Manual control of automatic vacuole selection [d]

Abbreviations: HE, hematoxylin and eosin; CNS, central nervous system

[a] The higher threshold was used in some sections due to weak HE staining or variation in section thickness.

[b] The threshold was deliberately set higher to decrease manual interference with the results, which may have excluded some smaller vacuoles and led to an underestimation of vacuole density.

[c] Closely connected vacuoles were counted as one by the software.

[d] False positives were manually deselected.

Supplemental Table S5. Summary of viral load in various regions of the central nervous system (CNS) across different age groups of pigs affected by congenital tremor (CT), as quantified by reverse transcription-quantitative PCR. Each group consists of five piglets (*n*=5).

Age Group	Area	Mean viral load (Cq)	95% CI (Cq)	Standard deviation
Newborns	Cerebrum	33.3	31.9 - 34.7	0.9
	Cerebellum	28.3	26.5 - 30.1	1.4
	Thoracic spinal cord	23.9	23.0 – 24.8	0.7
3-weeks-old	Cerebrum	33.1	31.1 – 34.7	1.6
	Cerebellum	22.3	19.4 - 25.2	2.3
	Thoracic spinal cord	26.0	22.4 – 39.5	2.8
4-5 months-old	Cerebrum	34.8	32.4 – 37.3	2.0
	Cerebellum	24.1	19.9 – 28.3	3.4
	Thoracic spinal cord	28.5	24.9– 32.1	2.9

Abbreviation: CI, confidence interval

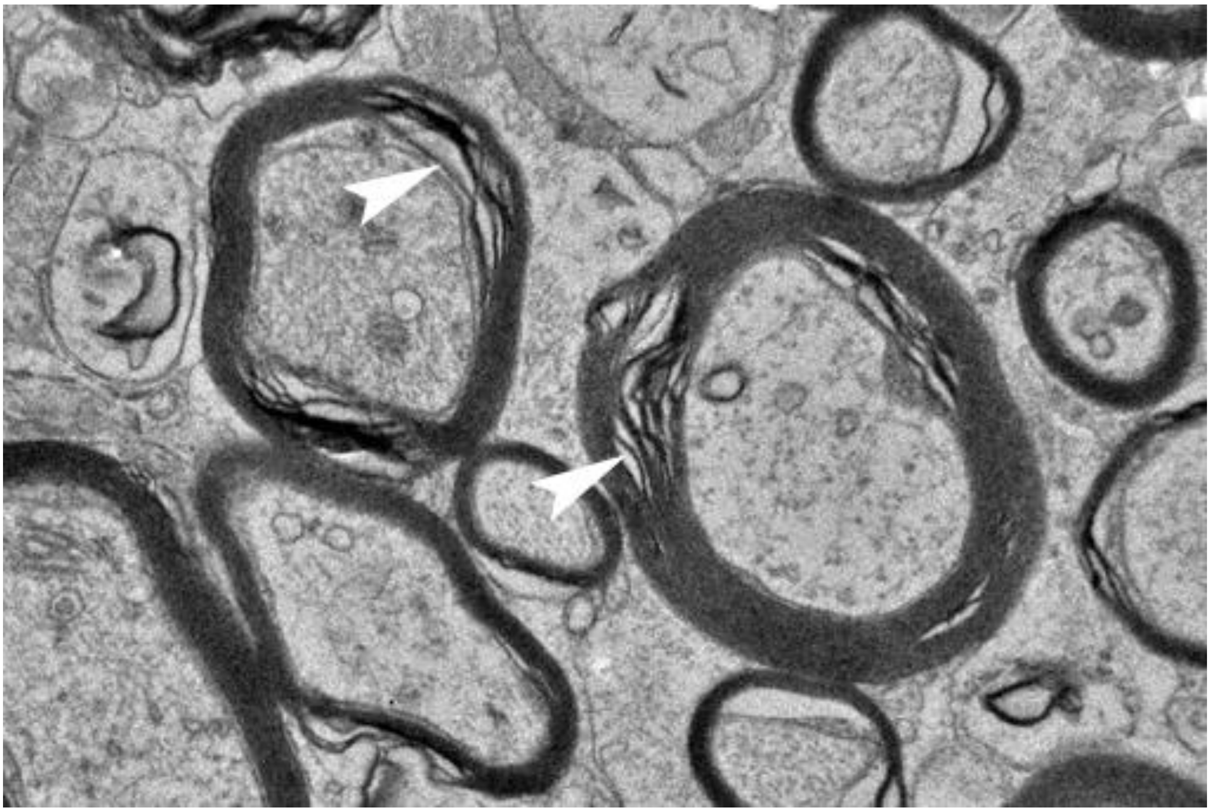

Supplemental Figure S1. Mild to moderate lamellar disruption of myelin sheaths (arrowheads), interpreted as fixation artifacts, observed in the cerebellar white matter of a 3-week-old pig affected by congenital tremor (CT). Transmission electron microscopy at 4000x magnification.
